# Gastric perforation repaired by hepatic round ligament: Case report and literature review

**DOI:** 10.1097/MD.0000000000050482

**Published:** 2026-09-04

**Authors:** Ribal Aby Hadeer, Angela Assaad, Hadi Farhat, Hany Maalouf, Oussama Chibly, Fidaa Alwan

**Affiliations:** a Department of General Surgery, University of Balamand, Beirut, Lebanon; b University of Balamand, Faculty of Medicine, Koura, Lebanon; c Department of General Surgery, Haykel Hospital, Tripoli, Lebanon.

**Keywords:** Gastric Cancer, Gastric perforation, Laparoscopy, Patch, Round ligament

## Abstract

**Rationale:**

Gastric perforation is a rare but life-threatening complication of gastric cancer, often presenting with acute peritonitis and requiring emergent surgical repair. The conventional treatment relies on an omental patch (Graham’s technique); however, this approach is not always feasible, particularly in patients with advanced disease or omental involvement.

**Patient concerns:**

We report the case of a 44-year-old male with advanced gastric adenocarcinoma complicated by peritoneal carcinomatosis who presented with acute abdomen following stent insertion.

**Diagnosis:**

Imaging confirmed gastric perforation and generalized peritonitis.

**Interventions:**

During emergent laparoscopic exploration, the omentum was unsuitable due to carcinomatous infiltration. Instead, the hepatic round ligament was mobilized and used as a patch to seal the perforation, achieving effective closure. The patient initially recovered well postoperatively; however, he later deteriorated due to progression of his underlying malignancy and died on postoperative day 16.

**Outcomes and lessons:**

This case highlights the hepatic round ligament as a viable alternative to the omentum in repairing gastric perforations, particularly in malignant or hostile abdominal environments. Wider application of this technique warrants further study to establish its safety and long-term outcomes.

## 1. Introduction

Gastric perforation is an acute surgical condition characterized by a complete rupture of the gastric wall, leading to a leakage of gastric fluid into the peritoneal cavity and thus resulting in peritonitis. Various conditions can cause such a full-thickness defect, including iatrogenic injuries, trauma, gastroduodenal ulcers, and malignancies. Regarding perforation in the setting of gastric cancer, it is an atypical but critical complication, frequently related to ulcerations, necrotic tumor tissue, or chemotherapy-related structural wall compromise. It occurs in approximately 0.4% of patients with gastric cancer and holds a significant mortality risk secondary to late diagnosis and defective functional capacity of these cancer patients.^[1]^

The surgical intervention for a perforated gastric wall is highly dependent on the primary cause and the clinical state of the patient. If the underlying cause is a malignancy, the surgical management can vary from directly sealing the defect using a Graham’s patch (an omental patch) to a more aggressive approach like partial or total gastrectomy along with lymph node dissection.^[2]^ It is reported that in some cases, the round ligament has been used successfully as a flap in sealing gastric and duodenal perforations when the classic omental patch technique was not possible^[3,4]^

## 2. Case report

This is the case of a 44-year-old male patient with a history of recurrent nephrolithiasis, with negative social and surgical history, who presented for diffuse abdominal pain associated with nausea and dysuria of 2 days duration. The patient reported vague abdominal pain that had increased in intensity for 2 days, associated with nausea and dysuria. He reported no episodes of vomiting, fever, chills, change in bowel habits, or loss of appetite. Laboratory investigations showed unremarkable CBC (Hb: 13.5, WBC:6.7, platelets: 307), CRP (8 mg/L), and unremarkable electrolytes and liver function tests. Urine analysis showed RBC 8–10/hpf, WBC: 2–4/hpf, and negative nitrites and leukocyte esterase.

**Figure 1.**
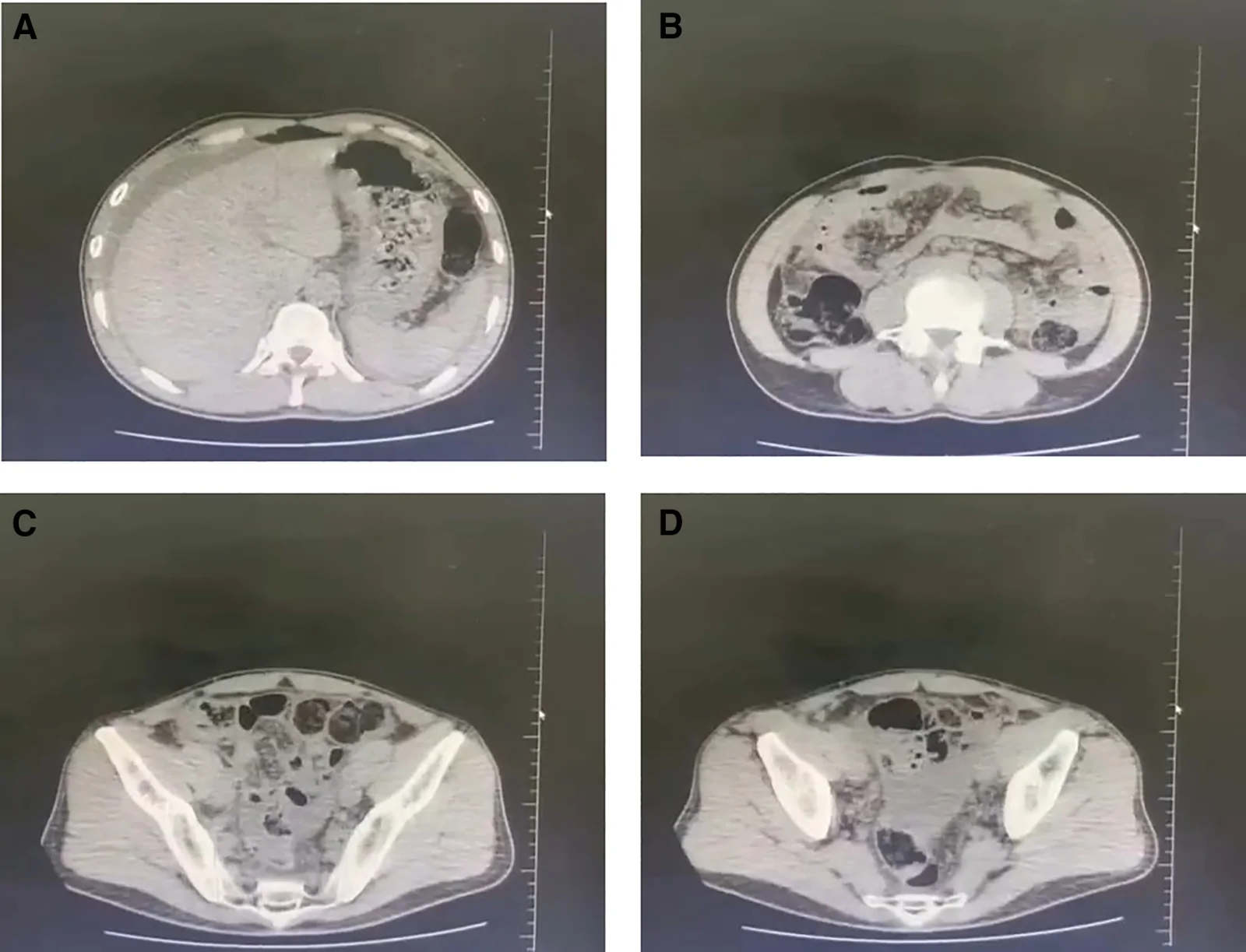
CT scan abdomen-pelvis.

Uroscan without IV contrast was done to rule out nephrolithiasis. It showed no renal or ureteral stones, no hydronephrosis, diffuse abdominal and pelvic ascites, and diffuse colonic fecal loading with segmental thickening of the sigmoid colon that could be due to peristaltic movement or neoplasm. Based on these results, a chest, abdomen, and pelvis CT scan with IV contrast was done to further investigate and rule out malignancy. It showed clear lungs with no worrisome nodules or mass, diffuse abdominal and pelvic ascites, diffuse fecal loading with no evidence of sigmoid tumor, diffuse omental infiltration with numerous sub-centimetric mesenteric nodular lesions, no evidence of hepatic lesions, and normal spleen, pancreas, and gallbladder (Fig. 1).

Patient underwent sigmoidoscopy, it was unremarkable with fecal stasis and no evidence of neoplastic lesions.

Given the normal sigmoidoscopy and unremarkable laboratory results, we opted for diagnostic laparoscopy with peritoneal biopsy to confirm the presence of peritoneal carcinomatosis. Laparoscopy showed diffuse peritoneal carcinomatosis that were confirmed by the biopsies taken. Patient’s symptoms worsened and started having episodes of vomiting, loss of appetite and increas in abdominal pain and distension. gastroscopy was carried out and showed diffuse gastritis with partially obstructive mass at the level of antrum, biopsies were taken and revelead gastric adenocarcinoma. multidiscplinary meeting was done and decision was made to insert gastric stent and start FOLFOX chemotherapy given the advanced stage of the cancer.

**Figure 2.**
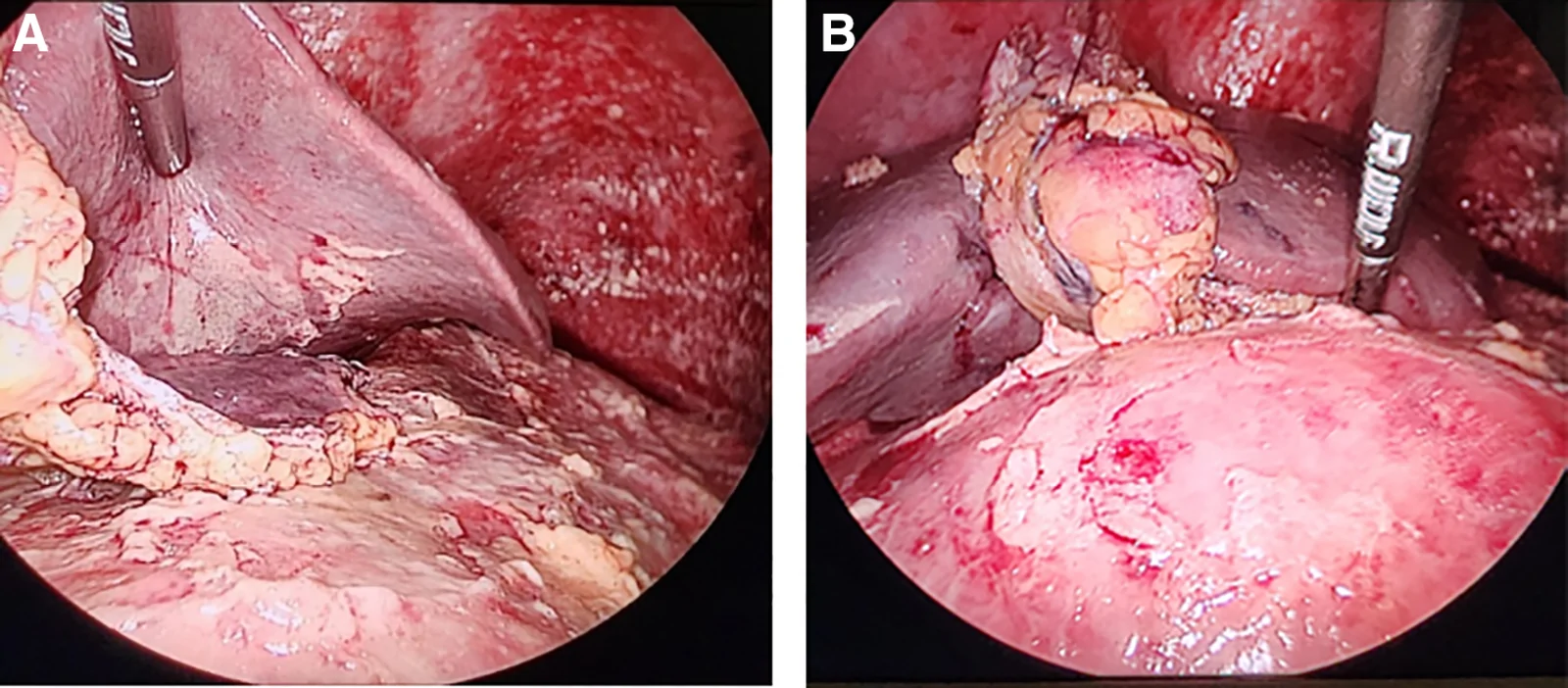
Gastric defect closed using round ligament.

Five days after stent insertion, the patient presented to the emergency department with diffuse abdominal pain, dyspnea, and altered general status. Vital signs were remarkable for hypotension (85/50 mmHg), tachycardia (110 bpm), and desaturation (SpO2: 86%). Laboratory tests were unremarkable. An urgent CT angiography of the chest, abdomen, and pelvis was done and showed bilateral filling defects in peripheral and central right and left pulmonary arteries compatible with extensive pulmonary embolisms, a distended stomach with the visualized stent in the gastric pylorus, and large anterior hydropneumoperitoneum suggestive of an underlying perforation.

patient was transferred to the OR for an emergent exploratory laparoscopy. Intraoperatively, generalized peritonitis was present, for which cultures were taken. An anterior antral perforation was encountered at the level of the gastric tumor next to the proximal end of the previously placed stent. Aggressive lavage with 3L normal saline was done, along with approximation of the edges of the perforation by 2 points Vicryl 2.0 sutures. It was impossible to utilize the omentum for omentoplasty due to the carcinogenic omental caking, so instead, the hepatic round ligament was released, and a gastrorrhaphy using the round ligament was done. Lamellated drains were inserted in the RUQ, RLQ, and Douglas pouch, and closure was done by layers (Fig. 2).

Postoperatively, the patient was transfered to ICU, started on therapeutic LMWH, antibiotics and TPN. patient was doing well, passed flatus day 3 post op and started progressive liquid diet, however; on day 13 post-op patient started experiencing several episodes of vomiting and abdominal pain and obstipation, so abdomen pelvis CT scan was done and showed bubbles in the anterior wall of pylorus with visualization of the gastric stent,partial opacification of the stomach and proximal duodenum, Distension of 2^nd^ duodenal segment reaching 6cm in diameter with tapered appearance of the 3^rd^ segment at the level of mesenteric clamp, likely due to SMA syndrome. The patient decided to stop any invasive procedure and signed DNI and DNR. On day 16 post-op the patient went into cardiorespiratory arrest and was pronounced dead.

## 3. Discussion

Gastric perforation, an infrequent aftereffect of peptic ulcer disease, malignancy, or trauma, is a life threatening surgical urgency. Immediate surgical intervention is required to repair and obliterate such gastrointestinal rupture to avert complications such as peritonitis and sepsis. The conventional surgical approach is the use of a piece of the omentum to seal the perforation, known as Graham’s patch. In the 1930s, Graham disclosed a case series where gastric perforations were successfully managed using the omentum as the reconstructing patch^[5]^

Yet, the omentum could be nonviable for use in some patients due to an underlying abnormal omental pathology, surgical history, or presence of adhesions. In such cases, an alternative approach is mandatory. One substitute is the use of the ligamentum teres hepatis (round ligament) as a reconstructing patch to cease the perforation.

The round ligament is a remnant of the umbilical vein that originates at the umbilicus where it ascends upwards to the anterosuperior surface of the liver^[6]^ and has a rich blood supply that originates from both the middle hepatic and inferior phrenic arteries.^[5]^ Fry et al and Munro et al were the first to use the falciform ligament to repair perforated peptic and duodenal ulcers respectively.^[5,7]^ This ligament has several assets when used in repairing gastric perforations. To start with, it is easily accessible in either laparoscopic or open surgery. Its fatty and fibrous tissue composition with fine vasculature^[5]^ reduces the chance of dehiscence or leakage and promotes healing. Furthermore, it also serves as a valid substitute if the omentum was defected and its usage would increase the risk for omental insufficiency. The hepatic round ligament is anatomically located away from most common perforation loci and would not be as heavily contaminated as the omentum in cases of peritonitis. This may make it a

### 3.1. Disinfected alternative in patching

To repair the perforated site laparoscopically, an umbilical camera port along with right and left midclavicular working ports must be inserted first. This will be followed by saline irrigation to localize the perforation site where either air bubbles or any leakage will be witnessed. The next step is to identify the ligamentum teres hepatis, which must then be ligated and dissected from the falciform ligament while carefully preserving its vascularity to obtain a wide flap. This resultant flap has an anterior adipose tissue surface and a peritoneal lining posterior surface. The flap will then be put in use as either a free graft or a patch. If ligated on both sides, it provides 12 to 23 cm of free graft with variable lumenal patency where it can then be sutured over the perforated site to strengthen and support its primary closure.^[8]^ This flap can also be further dissected longitudinally to create a patch and be pedicled or tunneled over the sutures to improve its healing process.^[7,9]^ The surgical technique was described in more detail by Baskaran V.^[8]^

In our case, the patient’s omentum was severely friable due to the presence of omental caking making it impossible to do Graham’s technique. Instead, the round ligament was mobilized and used successfully to close the gastric perforation at the anterior antrum of the stomach. There were no perioperative complications, and the patient was stable for 13 days until he started to deteriorate due to his advanced malignancy state that was beyond repair.

## 4. Conclusion

this laparoscopic intervention using the Round Ligament is effective in repairing perforations when conventional methods are not feasible, such as in our case. Although there is very limited literature about this approach, multiple case reports and small studies have described the success in sealing perforations using such a technique, decreasing postoperative complications and leaks with satisfactory outcomes.^[5,6]^ Although this technique is not yet adopted and widely used, it is a promising substitute, especially when the standard technique is not feasible to implement. Large case series and different comparative studies are required to further investigate its efficacy and long-term effects.

## Author contributions

**Conceptualization:** Ribal Aby Hadeer, Angela Assaad, Hany Maalouf.

**Writing—original draft:** Ribal Aby Hadeer, Angela Assaad, Hadi Farhat.

**Writing—review & editing:** Ribal Aby Hadeer, Hadi Farhat, Hany Maalouf, Oussama Chibly.

**Supervision:** Fidaa Alwan.

**Validation:** Fidaa Alwan.
